# Supplementary figures and images for: The association between the amino acid transporter LAT1, tumor immunometabolic and proliferative features and menopausal status in breast cancer
Source: PLoS One. 2023 Oct 11;18(10):e0292678. doi: 10.1371/journal.pone.0292678 (PMC10566702; doi:10.1371/journal.pone.0292678)

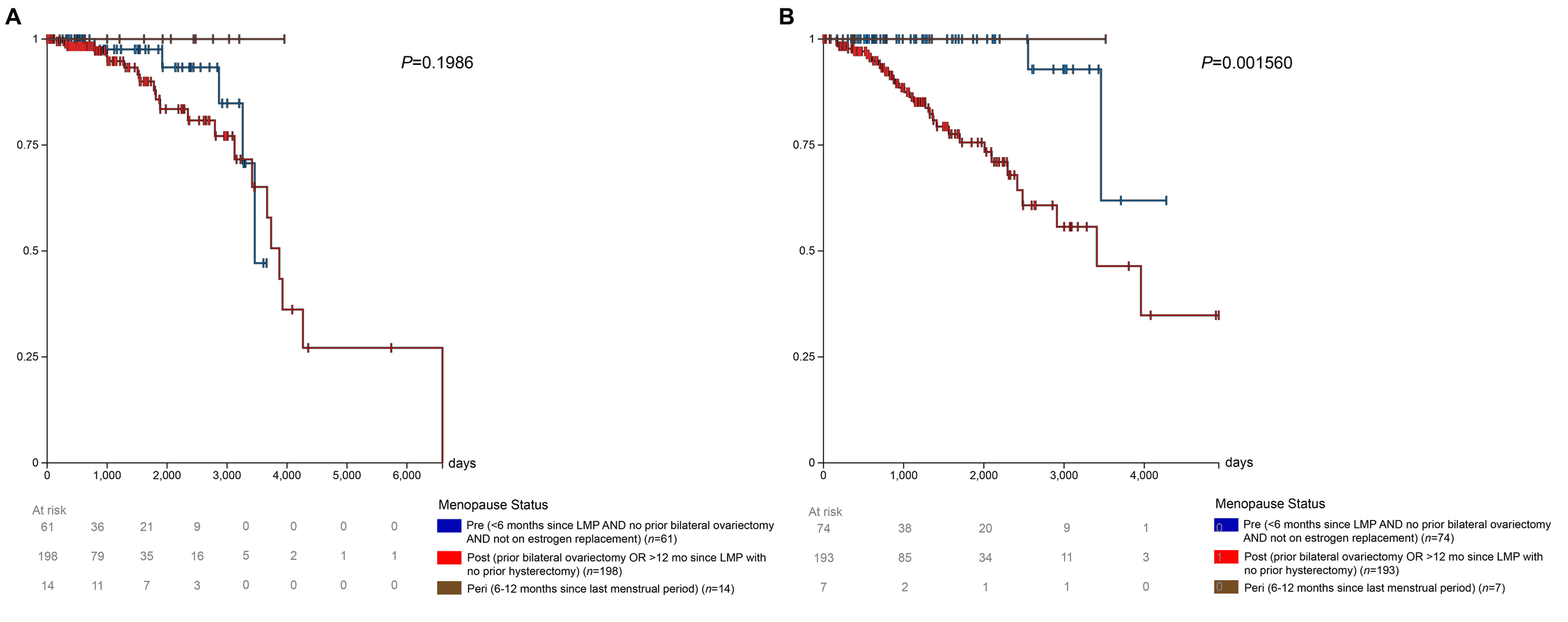

Supplement: S1 Fig — Patients were separated into (A) low (< 10.09 FPKM) and (B) high (> = 10.09 FPKM) LAT1 expression groups. Survival is observed for each menopausal status: premenopausal, postmenopausal, and peri-menopausal. (TIF) [file pone.0292678.s001.tif]

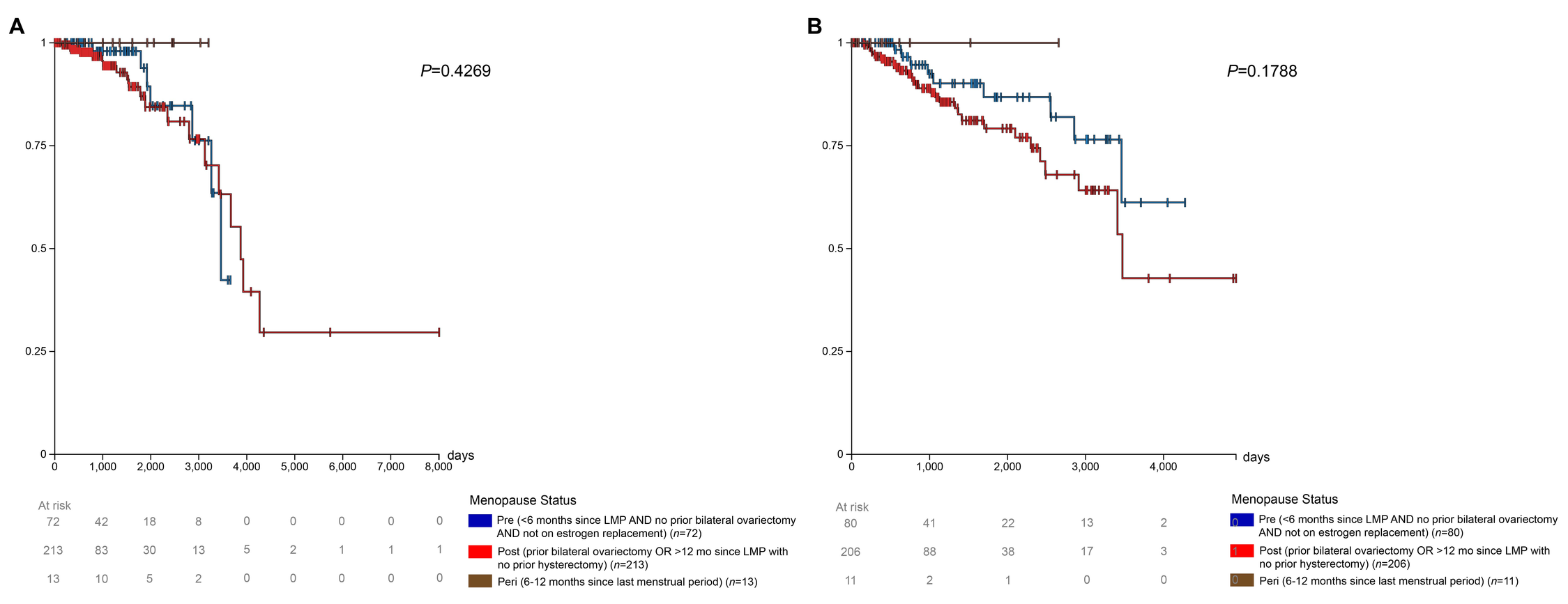

Supplement: S2 Fig — Patients were separated into (A) low (< 10.38 FPKM) and (B) high (> = 10.38 FPKM) expression groups. Survival is observed for each menopausal status: premenopausal, postmenopausal, and peri-menopausal. (TIF) [file pone.0292678.s002.tif]
