# Supplementary material for: The association between the amino acid transporter LAT1, tumor immunometabolic and proliferative features and menopausal status in breast cancer
Source: PLoS One. 2023 Oct 11;18(10):e0292678. doi: 10.1371/journal.pone.0292678 (PMC10566702; doi:10.1371/journal.pone.0292678)
Supplement: S1 Table — P-values are shown. (DOCX) [file pone.0292678.s003.docx]

|  | Premenopausal Patients | Postmenopausal Patients |
| --- | --- | --- |
| SUV_Mean_ | 0.959 | 0.291 |
| SUV_Peak_ | 0.926 | 0.528 |
| SUV_Max_ | 0.992 | 0.436 |
