# Supplementary material for: The association between the amino acid transporter LAT1, tumor immunometabolic and proliferative features and menopausal status in breast cancer
Source: PLoS One. 2023 Oct 11;18(10):e0292678. doi: 10.1371/journal.pone.0292678 (PMC10566702; doi:10.1371/journal.pone.0292678)
Supplement: S2 Table — (DOCX) [file pone.0292678.s004.docx]

|  | Premenopausal Patients | Postmenopausal Patients |
| --- | --- | --- |
| SUV_Mean_ | 0.735 | **0.004** |
| SUV_Peak_ | 0.268 | 0.157 |
| SUV_Max_ | 0.437 | 0.173 |
| Ki-67 | 0.205 | 0.993 |
