# Supplementary material for: The association between the amino acid transporter LAT1, tumor immunometabolic and proliferative features and menopausal status in breast cancer
Source: PLoS One. 2023 Oct 11;18(10):e0292678. doi: 10.1371/journal.pone.0292678 (PMC10566702; doi:10.1371/journal.pone.0292678)
Supplement: S3 Table — The log2 fold change of each parameter was compared. The comparisons’ p-values are shown above. Shapiro-Wilk tests were used to assess the data’s normality. Based on these results, normally distributed data were compared using the Student’s t-test, and all other analyses used the Mann-Whitney test. a These comparisons use the Student’s t-test. (DOCX) [file pone.0292678.s005.docx]

|  | Basophils | Eosinophils | Neutrophils | Monocytes | Lymphocytes |
| --- | --- | --- | --- | --- | --- |
| SUV_Mean_ | 0.339 | 0.619 | 0.213 | 0.184 | 0.115 |
| SUV_Peak_ | 0.339 | 0.803 | 0.319 | 0.229 | 0.147 |
| SUV_Max_ | 0.318 | 0.803 | 0.431 | 0.340 | 0.171 |
| Ki-67 | 0.755 ^a^ | 0.901 | 0.135 | 0.584 ^a^ | 0.245 |
