# Supplementary material for: The association between the amino acid transporter LAT1, tumor immunometabolic and proliferative features and menopausal status in breast cancer
Source: PLoS One. 2023 Oct 11;18(10):e0292678. doi: 10.1371/journal.pone.0292678 (PMC10566702; doi:10.1371/journal.pone.0292678)
Supplement: S4 Table — The log2 fold change of each parameter was compared. The comparisons’ p-values are shown above. Significant results are bolded, and marginally significant results are bolded and italicized. (DOCX) [file pone.0292678.s006.docx]

|  | Basophils | Eosinophils | Neutrophils | Monocytes | Lymphocytes |
| --- | --- | --- | --- | --- | --- |
| SUV_Mean_ | **<0.001** | **<0.001** | 0.235 | **<0.001** | **<0.001** |
| SUV_Peak_ | **<0.001** | **<0.001** | 0.187 | **<0.001** | **<0.001** |
| SUV_Max_ | **<0.001** | **<0.001** | 0.124 | **<0.001** | **<0.001** |
| Ki-67 | **0.010** | **<0.001** | ***0.091*** | **0.002** | **<0.001** |
