# Supplementary material for: The association between the amino acid transporter LAT1, tumor immunometabolic and proliferative features and menopausal status in breast cancer
Source: PLoS One. 2023 Oct 11;18(10):e0292678. doi: 10.1371/journal.pone.0292678 (PMC10566702; doi:10.1371/journal.pone.0292678)
Supplement: Attachment — Submitted filename: Plos One comments.docx [file pone.0292678.s007.docx]

In this article, using transcriptomic data analysis, the authors demonstrate that high tumor LAT1 expression predicts abbreviated survival in postmenopausal breast cancer patients compared to peri- or premenopausal patients. The authors also discuss about tumor immunometabolism and proliferation and show that these features are associated with pre and postmenopausal statuses of breast cancer patients. They show that tumor Ki-67 staining significantly and positively correlated with 18F-FLT uptake in postmenopausal patients.

LAT1 expression is already well established as an independent poor prognostic factor in invasive breast cancer and other cancer types. While there are many shortcomings in the article which makes conclusions rather confounding, like the use of BMI as an obesity marker and including weak insignificant correlations of immune cells with SUV, and Ki-67, the correlation of LAT1 expression with the menopausal status is clinically relevant and significant. However, the lack of inclusion of other molecular factors in this study raises concerns, see below, and undermines the clinical association and utility of this work.

1. The authors do not show if LAT1 expression correlates with menopausal status in breast cancer subtypes especially in ER+, and HER2- tumors. There is a huge body of literature suggesting LAT1 as an estrogen responsive gene and that LAT1 is responsible for endocrine therapy resistance. Likewise, HER2- tumors have been shown to have high expression of LAT1 and do not respond to current therapies. It would be interesting to see if LAT1 expression can stratify pre and post-menopausal breast cancer patient survival in these subtypes. If so, these cohorts can benefit from drugs like JPH203, currently in clinical trial, targeting LAT1 activity.
2. Postmenopausal patients have increased systemic circulation of inflammatory cytokines such as IL-6, IL-1beta, and TNFa. Coincidently, these cytokines are also known to regulate the expression of LAT1 and can promote tumor-immune infiltration. The authors do not show if any of these post-menopausal cytokines correlate with LAT1 expression in the same data sets as a casual mechanistic link for LAT1 dependent abbreviated survival? If so, this rationally explains the significant worse survival association of LAT1 in postmenopausal patients.
3. Both Ki-67 and 81F-FLT uptake are good quantitative markers of tumor proliferation. The authors show a strong association of Ki-67 with 18F-FLT uptake in postmenopausal patients. Does Ki-67 and 18F-FLT uptake show a significantly positive association with LAT1 expression in postmenopausal patients in the same dataset? This is because LAT1 is known to activate mTORC1 through uptake of leucine and mTOR activation drives cell proliferation.
4. In the title, the authors declare that the relationships between tumor immunometabolic and proliferative features depend on the menopausal status in breast cancer. While the metabolic and proliferative features do significantly correlate with menopausal status, the immune cells demonstrate only weak correlation and is not significant. The weak positive correlation in postmenopausal patients could again be because of the high levels of systemic cytokines in these cohort.

The authors need to address these concerns to strengthen the use of LAT1 as a clinical biomarker for breast cancer patient prognosis.
