# Supplementary material for: The association between the amino acid transporter LAT1, tumor immunometabolic and proliferative features and menopausal status in breast cancer
Source: PLoS One. 2023 Oct 11;18(10):e0292678. doi: 10.1371/journal.pone.0292678 (PMC10566702; doi:10.1371/journal.pone.0292678)
Supplement: Attachment — Submitted filename: LAT1 rebuttal 2.docx [file pone.0292678.s009.docx]

We thank the reviewers and editors for their time spent evaluating this manuscript. We are delighted that the reviewers and editor find the manuscript of interest, and that Reviewer 1 feels that we have addressed all of their comments. We have edited the manuscript in response to Reviewer 2’s remaining comments as described below.

**Reviewer #2: The authors have addressed the concerns arisen from data analysis and interpretation. First, statistical analysis in this manuscript were revised by the authors, remaining a few concerns that all studies have. The authors explained properly on the analysis. Second, the authors revised the ambiguous medical interpretations by describing clearly and adding the limitations and shortcomings about their result and interpretation. This study suggests that LAT1 expression status can be a possible biomarker in breast cancer patients. They also suggested a few clinical factors that highly related to breast cancer incidence such as a proliferation status, obesity, immune / inflammation status. Despite the limitation of available clinical data representing the risk factors, the authors demonstrated the best way predicting the prognosis of breast cancer patients with factors including LAT1 expression. Since the authors mentioned the necessity of future studies to validate this finings, the readers can interpret and assume the intention and purpose of this article. The article also suggested the correlation among the factors before / after menopause, indicating this article has a novelty in this field. In addition, this article is of importance to describe the method and result of early biomarker study in breast cancer research. I recommend to publish this article with the minor revision.**

We greatly appreciate the reviewer’s many positive comments on our revision, indicating that we have addressed most of the issues they raised. We are delighted that the reviewer believes “this article is of importance to describe the method and result of early biomarker study in breast cancer research.” We have revised according to each of the reviewer’s comments as described below, and after these revisions hope the article will be deemed acceptable for publication.

**1. The purpose of Figure 1 is to show the association between two factors. Is it necessary to show how much different between two factors in Supple Table 5-8? Also, the authors performed the U-test (non-parametric) instead of T-test (parametric), however in Figure 1, the authors performed the pearson test (parametric) instead of spearman (non-parametric) with the same data. In my suggestion, Table 5-8 is not necessary.**

We thank the reviewer for these comments, and in accordance, have removed Supplemental Tables 5-8. We agree that considering the presentation of data in Figure 1, Supplemental Tables 5-8 are not necessary.

**2. I think it's better to describe more details in the legend for figure 8 . Please add conclusions or explains with the fig 8 legend title for the readers who read the summary figure first.**

We have added additional description as follows: “The current study analyzed ^18^F-FLT uptake, immune infiltrate levels, body weights, LAT1 expression, and tumor proliferative factors observed in breast cancer patients.”
